# Supplementary material for: The influence of hydrogel stiffness on axonal regeneration after spinal cord injury
Source: PLoS One. 2025 Jun 25;20(6):e0325798. doi: 10.1371/journal.pone.0325798 (PMC12192028; doi:10.1371/journal.pone.0325798)
Supplement: S1 Table — (DOCX) [file pone.0325798.s001.docx]

**Table S1 Antibody Catalog**

| **IF antibody** | **company** | **catalog number** | **dilution** |
| --- | --- | --- | --- |
| rabbit anti-NF | Cell Signaling Technology | 30564 | 1:400 |
| goat anti-GFAP | Abcam | Ab53554 | 1:400 |
| Donkey Anti-Rabbit IgG H&L (Alexa Fluor® 488) | Abcam | ab150073 | 1:400 |
| Donkey Anti-Goat IgG H&L (Alexa Fluor® 488) | Abcam | ab150132 | 1:400 |
